# Identification of Spacer and Protospacer Sequence Requirements in the Vibrio cholerae Type I-E CRISPR/Cas System

**DOI:** 10.1128/mSphere.00813-20

**Published:** 2020-11-18

**Authors:** Jacob Bourgeois, David W. Lazinski, Andrew Camilli

**Affiliations:** a Department of Molecular Biology and Microbiology, Graduate School of Biomedical Sciences, Tufts University, School of Medicine, Boston, Massachusetts, USA; University of Iowa

**Keywords:** CRISPR/Cas, *Vibrio cholerae*, protospacer-adjacent motif

## Abstract

The prokaryotic adaptive immune system CRISPR/Cas serves as a defense against bacteriophage and invasive nucleic acids. A type I-E CRISPR/Cas system has been detected in classical biotype isolates of Vibrio cholerae, the causative agent of the disease cholera. Experimental characterization of this system revealed a functional immune system that operates using a 5′-TT-3′ protospacer-adjacent motif (PAM) for interference. However, several designed spacers against the 5′-TT-3′ PAM do not interfere as expected, indicating that further investigation of this system is necessary. In this study, we identified additional conserved sequences, including a pyrimidine in the 5′ position of the spacer and a purine in the complementary position of the protospacer using 873 unique spacers and 2,267 protospacers mined from CRISPR arrays in deposited sequences of V. cholerae. We present bioinformatic evidence that during acquisition the protospacer purine is captured in the prespacer and that a 5′-RTT-3′ PAM is necessary for spacer acquisition. Finally, we demonstrate experimentally, by designing and manipulating spacer and cognate PAMs in a plasmid conjugation assay, that a 5′-RTT-3′ PAM is necessary for CRISPR interference, and we discover functional consequences for spacer efficacy related to the identity of the 5′ spacer pyrimidine.

**IMPORTANCE** Bacterial CRISPR/Cas systems provide immunity by defending against phage and other invading elements. A thorough comprehension of the molecular mechanisms employed by these diverse systems will improve our understanding of bacteriophage-bacterium interactions and bacterial adaptation to foreign DNA. The Vibrio cholerae type I-E system was previously identified in an extinct classical biotype and was partially characterized for its function. Here, using both bioinformatic and functional assays, we extend that initial study. We have found that the type I-E system still exists in modern strains of V. cholerae. Furthermore, we defined additional sequence elements both in the CRISPR array and in target DNA that are required for immunity. CRISPR/Cas systems are now commonly used as precise and powerful genetic engineering tools. Knowledge of the sequences required for CRISPR/Cas immunity is a prerequisite for the effective design and experimental use of these systems. Our results greatly facilitate the effective use of one such system. Furthermore, we provide a publicly available software program that assists in the detection and validation of CRISPR/Cas immunity requirements when such a system exists in a bacterial species.

## Abstract (precis)

Bacterial CRISPR/Cas systems provide immunity by defending against phage and other invading elements. A thorough comprehension of the molecular mechanisms employed by these diverse systems will improve our understanding of bacteriophage-bacterium interactions and bacterial adaptation to foreign DNA. The Vibrio cholerae type I-E system was previously identified in an extinct classical biotype and was partially characterized for its function. Here, using both bioinformatic and functional assays, we extend that initial study. We have found that the type I-E system still exists in modern strains of V. cholerae. Furthermore, we defined additional sequence elements both in the CRISPR array and in target DNA that are required for immunity. CRISPR/Cas systems are now commonly used as precise and powerful genetic engineering tools. Knowledge of the sequences required for CRISPR/Cas immunity is a prerequisite for the effective design and experimental use of these systems. Our results greatly facilitate the effective use of one such system. Furthermore, we provide a publicly available software program that assists in the detection and validation of CRISPR/Cas immunity requirements when such a system exists in a bacterial species.

## INTRODUCTION

Clustered regularly interspaced short palindromic repeats (CRISPR) and CRISPR-associated (*cas*) genes comprise an adaptive immune system found in many bacteria and archaea that protects cells from invasion by foreign nucleic acid (1). These CRISPR/Cas systems consist of several *cas* genes that encode proteins for adaptation and immunity alongside an array of short repeat sequences alternating with sequences derived from foreign invaders, termed spacers. CRISPR targets and degrades invading nucleic acid in a sequence-specific manner. Most systems protect against DNA, although rarer types also target RNA (2, 3). In adaptation, the CRISPR/Cas system acquires and stores novel invading DNA sequences as spacers in its array. Novel spacers are integrated at the 5′ end of the array immediately downstream from the leader-proximal repeat (1, 4). In immunity, the CRISPR locus, which contains recorded spacers, is first transcribed and processed to yield CRISPR RNAs (crRNAs). Upon recognition by a crRNA of a complementary DNA sequence called the protospacer, the target DNA is cleaved, thereby providing immunity.

There is substantial diversity among CRISPR/Cas systems in architecture, protein composition, target affinity, and mechanisms of interference and adaptation. Systems are broadly classified into multisubunit effector complexes in class I and single-unit effectors in class II (3). These classes are further organized into several types and subtypes by virtue of genetic organization and Cas protein composition (3, 5, 6). Aside from complementarity between spacer and protospacer, CRISPR/Cas systems possess an additional sequence requirement adjacent to the protospacer called a protospacer-adjacent motif, or PAM (7, 8). The PAM size and location varies by CRISPR system and is typically a 2- to 5-bp sequence that may be located upstream or downstream of the target (6, 9, 10). An intact PAM is essential for acquisition and interference; mutations in this motif provide escape from CRISPR-mediated immunity (11). In the CRISPR array, the repeat sequence that is adjacent to the spacer lacks a PAM. This absence prevents autoimmunity, since the crRNA is unable to cleave the DNA template from which it is transcribed.

Vibrio cholerae is a Gram-negative facultative bacterium that is the causative agent of cholera (12, 13). The seventh and most recent pandemic began in 1961, caused by V. cholerae serotype O1 biotype El Tor, which replaced the classical biotype of previous pandemics (12, 13). A type I-E CRISPR/Cas system has recently been described in the now-extinct classical biotype of V. cholerae. Genomic island GI-24, found in the classical strain O395, was predicted to encode Cas proteins (14). Box et al. introduced GI-24 into the El Tor biotype and demonstrated experimentally that the putative CRISPR/Cas system is functional and that artificial induction of the system provides resistance against virulent bacteriophage under laboratory conditions (15). Based on sequence alignment of 33 cognate protospacers from spacers mined from five sequenced classical isolates and analysis of bacteriophage escape mutants, the PAM for the system was determined to be 5′-TT-3′, located immediately downstream of the protospacer.

A useful application of the CRISPR/Cas system is the creation of insertions, deletions, and point mutations in virulent bacteriophages, manipulations that can be quite difficult using conventional genetic engineering techniques (16, 17). By introducing spacers against wild-type sequences into a host strain containing *cas* genes, mutant bacteriophage are enriched as they escape CRISPR/Cas immunity. Such escape mutants can be precisely edited by the provision of homologous recombination templates that contain the desired alteration (16, 17). This principle has been used to edit bacteriophages in several model systems, such as Escherichia coli and Streptococcus thermophilus, with editing efficiencies as high as 100% (18, 19). In the characterization of the type I-E CRISPR/Cas system in V. cholerae, Box et al. placed a minimal CRISPR array onto a plasmid such that engineered spacers could be introduced easily for the editing of bacteriophage (15). Although all of the engineered spacers functioned upon overexpression of the *cas* genes and CRISPR array, the degree of interference varied by 4 orders of magnitude (15). This variation in interference suggests that there are further unknown parameters that impact the efficiency of interference in the V. cholerae CRISPR/Cas system.

Knowledge of the parameters for interference is crucial in the design of effective spacers for CRISPR/Cas. Furthermore, dissection of CRISPR/Cas systems expands our knowledge of the broader molecular mechanisms of CRISPR adaptation and interference. In this study, we greatly expand the database of spacers and cognate protospacers by mining deposited sequences of V. cholerae with identical type I-E CRISPR array repeats and identify a conserved pyrimidine on the 5′ end of naturally acquired spacers that is derived from the incoming prespacer. Using the established plasmid-based CRISPR system, we determined experimentally that this pyrimidine is not required for efficient interference but that its complementary purine in target DNA is required, and we therefore propose a 5′-RTT-3′ PAM. Finally, we observed that a cytidine in the 5′ end of the spacer reduces the efficiency of interference even in the absence of potential base pairing with the PAM.

(This article was submitted to an online preprint archive [20]).

## RESULTS

### Analysis of CRISPR repeats in publicly available sequences of V. cholerae identifies a conserved 5′-pyrimidine in spacers.

**FIG 1:**
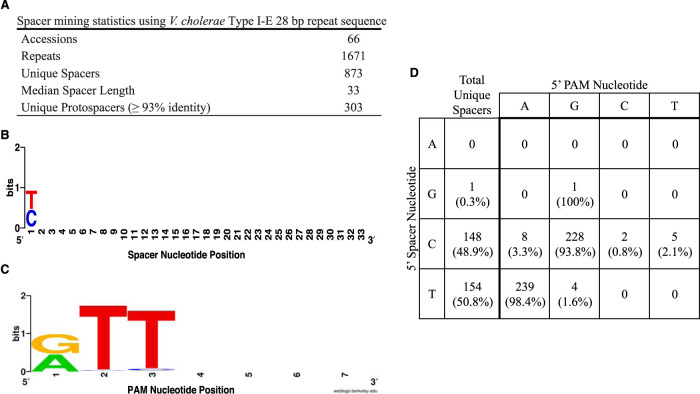
Analysis of spacer and protospacer-adjacent motifs mined from deposited V. cholerae sequences. (A) Overview of spacer mining statistics obtained from detection of repeats. (B) Sequence alignment of unique spacers derived from perfect repeats, shown in the 5′-to-3′ direction. For spacers longer than 33 bp, only the first 33 bp were used in the creation of the Weblogo. (C) Sequence alignment of the PAM retrieved immediately downstream from protospacers, shown in the 5′-to-3′ direction. The first position of the PAM is aligned with the 5′ position of the spacer. (D) Frequency table of 5′ spacer and 5′ PAM nucleotide identity for 487 unique spacer-protospacer pairs. “Total unique spacers” refers to the number of unique spacers with detected cognate protospacers that begin with the specific nucleotide.

The original characterization of the type I-E CRISPR/Cas system in V. cholerae identified 78 unique spacers and 22 corresponding protospacers across five sequenced isolates (15). To obtain more spacers and protospacers, we identified additional CRISPR-containing V. cholerae isolates by querying NCBI databases for strains containing the 28-bp V. cholerae O395 CRISPR repeat (5′-GTCTTCCCCACGCAGGTGGGGGTGTTTC-3′). In total, 1,671 repeats were detected over 66 sequence accessions containing 45 unique strains isolated over a wide geographical area (Fig. 1A; see also Table S1 in the supplemental material). Of note, several isolates were obtained as recently as 2018, indicating that the CRISPR/Cas system first identified in the extinct classical biotype itself is not extinct and continues to influence modern V. cholerae genomes. From these samples, spacers and protospacers were extracted using a custom script written using the programming language Python (see Materials and Methods). To ensure validity, only spacers that were no longer than 50 nucleotides, were flanked by perfect repeat sequences, and did not contain ambiguous nucleotides were considered. Only protospacers that were at least 93% identical over 96% of the corresponding spacer were considered. Overall, our method obtained 873 unique spacers (Fig. 1A; Table S2). The median length of spacer sequences was 33 bp, in agreement with the previous characterization (15). Several spacers are shared among analyzed isolates; a spacer targeting vibriophages Rostov 7 and X29 is seen in eight separate isolates. However, nearly 75% of spacers are unique to a single strain, suggesting separate encounters and acquisition events (Table S3).

10.1128/mSphere.00813-20.2 TABLE S1 Overview of deposited V. cholerae sequences harboring the type I-E CRISPR/Cas 28-bp repeat sequence. Download Table S1, XLS file, 0.04 MB. Copyright © 2020 Bourgeois et al. 2020 Bourgeois et al. This content is distributed under the terms of the Creative Commons Attribution 4.0 International license.

10.1128/mSphere.00813-20.3 TABLE S2 Overview of mined spacers from identified V. cholerae sequences harboring the type I-E CRISPR/Cas system. Download Table S2, XLS file, 0.3 MB. Copyright © 2020 Bourgeois et al. 2020 Bourgeois et al. This content is distributed under the terms of the Creative Commons Attribution 4.0 International license.

10.1128/mSphere.00813-20.4 TABLE S3 Frequency of mined spacers in identified V. cholerae sequences harboring the type I-E CRISPR/Cas system. Download Table S3, XLS file, 0.2 MB. Copyright © 2020 Bourgeois et al. 2020 Bourgeois et al. This content is distributed under the terms of the Creative Commons Attribution 4.0 International license.

Of the 873 unique spacers, 303 (34%) matched at least one valid protospacer target (Fig. 1A and D; Table S3). In accordance with the role of CRISPR as a bacterial immune system, several protospacers from many diverse bacteriophages were identified, including vibriophages fs1 and fs2, kappa, and CP-T1, among others, for a total of 2,267 protospacers. In several cases, for individual unique spacers, we found nucleotide polymorphisms between cognate protospacer sequences obtained from different organisms. Overall, we obtained 487 unique spacer-protospacer pairs (Fig. 1D; Table S4). Additional protospacers mapped to prophages found in other *Vibrio* isolates, environmental broad-host-range replicons such as repSD172 (21), or identical spacers in sequenced CRIPSR arrays. In total, our analysis expanded the list of unique spacers acquired naturally by >10-fold and identified hundreds of novel target protospacers and genetic elements. Furthermore, the recent isolation dates of many isolates suggest that CRISPR/Cas is still an active force in V. cholerae evolution. However, no isolates of the V. cholerae El Tor biotype, the causative agent of the current, seventh cholera pandemic (12), were detected.

10.1128/mSphere.00813-20.5 TABLE S4 Overview of mined protospacers from identified V. cholerae sequences harboring the type I-E CRISPR/Cas system detected by BLAST to the NCBI nonredundant database. Download Table S4, XLS file, 1.0 MB. Copyright © 2020 Bourgeois et al. 2020 Bourgeois et al. This content is distributed under the terms of the Creative Commons Attribution 4.0 International license.

Upon further inspection of mined data, nearly every extracted spacer (99.7% of 303 unique spacers with cognate protospacers) began with a pyrimidine in the 5′ position (Fig. 1D; Table S2). Alignment of unique spacer sequences confirmed that the 5′ position of the spacer is a conserved pyrimidine (Fig. 1B). To detect the presence of the protospacer-adjacent motif (PAM), we aligned mined protospacers, starting from the 3′-most nucleotide of the protospacer that aligns with the 5′ spacer nucleotide. Alignment revealed a strong 5′-RTT-3′ motif, where the conserved purine is complementary to the 5′ pyrimidine of the spacer (Fig. 1C). Additionally, examination of 483 unique spacer-protospacer pairs reveals that spacers with a 5′ cytidine have a cognate 5′ PAM guanosine 93.8% of the time, and 98.4% of those matching 5′ thymidine spacers possess a 5′ adenosine PAM. This near-perfect complementarity observed between the purine of the PAM and the pyrimidine of the first position of the spacer provides evidence that the purine of the PAM is captured during spacer acquisition. The small number of cases where complementarity is lacking presumably result from mutational escape from CRISPR targeting. Therefore, our bioinformatic results suggest a role for the purine in capture and adaptation, and thus, the functional PAM may be 5′-RTT-3′ instead of 5′-TT-3′.

### The conserved spacer pyrimidine and complementary protospacer purine are necessary for CRISPR interference.

**FIG 2:**
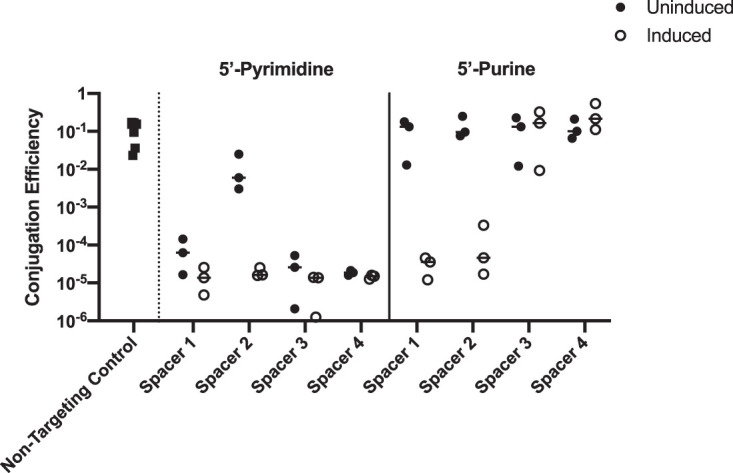
Targeting activities of 5′-pyrimidine and 5′-purine spacers against 5′-RTT-3′ and 5′-YTT-3′ PAMs in *aad9*, respectively. Shown is the conjugation efficiency of mating pDL1301, a conjugatable plasmid containing the *aad9* gene, into V. cholerae El Tor possessing Cascade and engineered targeting spacers on a plasmid-based pCRISPR (filled circles). The nontargeting control contains pCRISPR, encoding a spacer with no homology to pDL1301 (filled squares). Open circles show the effect of induction of Cascade and crRNA with 100 μM IPTG on interference. Data represent the conjugation efficiency of a plasmid into three independently obtained V. cholerae exconjugates.

PAM sequences are required for both acquisition and interference (9, 10, 22). Thus far, we have provided bioinformatic evidence consistent with a 5′-RTT-3′ PAM in the target DNA being required for acquisition. To experimentally determine the importance of the 5′ spacer nucleotide and 5′-RTT-3′ PAM in CRISPR interference, we designed eight independent spacers targeted against the *aad9* spectinomycin adenyltransferase gene, four of which have a 5′-pyrimidine against a 5′-RTT-3′ PAM, and four of which have a 5′-purine against a 5′-YTT-3′ PAM (Table 1). We introduced these spacers into a functional plasmid-based inducible CRISPR/Cas targeting system, pCRISPR (15). These eight targeting plasmids were then introduced into AC6625, a V. cholerae El Tor strain containing inducible *cas* genes from classical isolate O395. We then tested the efficiency of conjugation of pDL1301, a plasmid containing the *aad9* gene, into each targeting strain. The *trp*-*lac* fusion promoter (*tac*) is used to drive the expression of both crRNA and Cas proteins in the targeting strains (15). The *tac* promoter is known to function at modest levels in the absence of isopropyl-β-d-thiogalactopyranoside (IPTG) and at very high levels upon induction (23). Initial experiments were therefore performed in the absence of IPTG. The efficiency of conjugation of pDL1301 into a strain with a nontargeting spacer (5′-TGAGACCAGTTCTCTCGGAAGCTCAAAGGTCTC-3′) was obtained as a control. All four 5′-pyrimidine spacers provided appreciable interference against their 5′-RTT-3′ PAMs, three of which had conjugation efficiencies 10^3^- to 10^4^-fold lower than that of the nontargeting control (Fig. 2). In contrast, all four 5′-purine spacers had efficiencies no better than the control at targeting 5′-YTT-3′ PAMs. These results demonstrate that either a 5′-pyrimidine in the spacer or a 5′-purine in the PAM, or both, are needed to elicit interference.

**TABLE 1:** Engineered spacers against the *aad9* gene in the target plasmid

Name	Targeting strand	PAM	Spacer sequence
Pyrimidine-1	–1	5′-ATT-3′	5′-TGGTTCAGATACGACGACTAAAAAGTCAAGATC-3′
Pyrimidine-2	1	5′-GTT-3′	5′-CGAAAAAATAAAAGAATATACGGAAATTATGAC-3′
Pyrimidine-3	–1	5′-ATT-3′	5′-TGGAATATCAGGTAGTAATTCCTCTAAGTCATA-3′
Pyrimidine-4	–1	5′-ATT-3′	5′-TATAGAGTTGGTTTCATCATCCTGATAATTATC-3′
Purine-1	1	5′-TTT-3′	5′-AGAATATACGGAAATTATGACTTAGAGGAATTA-3′
Purine-2	–1	5′-TTT-3′	5′-AGTTAATATAGAGTTGGTTTCATCATCCTGATA-3′
Purine-3	–1	5′-TTT-3′	5′-AATCATACGGCATAAAGTTAATATAGAGTTGGT-3′
Purine-4	–1	5′-TTT’3′	5′-AATTCTCTCCCTATGTTCTAATGGAGAAGATTC-3′

To investigate the effect of induction of targeting machinery on conjugation efficiency, conjugation was also performed in the presence of IPTG on the selection plate. Induction was able to rescue the weak targeting activity of 5′-pyrimidine spacer 2 to the same level as those of other 5′-pyrimidine spacers (Fig. 2). Additionally, induction was able to elicit targeting in 5′-purine spacers 1 and 2, but not 3 and 4. These results suggest that induction of both Cascade and crRNA may rescue poorly targeting spacers, a phenomenon observed in other CRISPR systems (24, 25).

### Transversion mutation at the +1 targeting position reverses interference efficacy.

**FIG 3:**
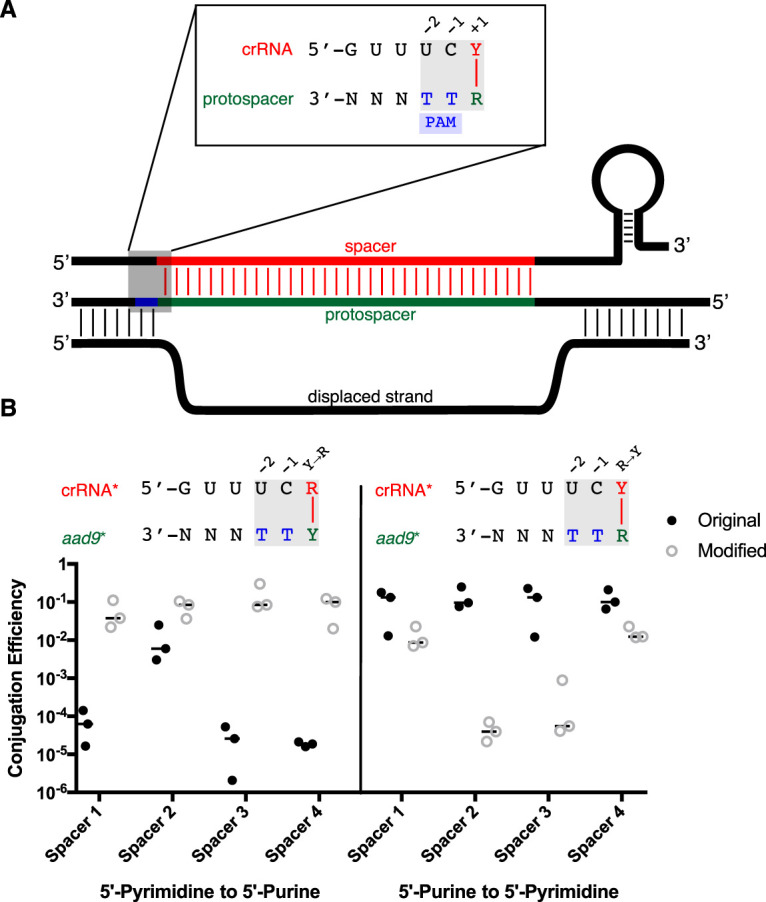
Mutating the 5′ spacer nucleotide and the corresponding *aad9* target PAM reverses crRNA targeting efficacy. (A) Schematic of crRNA strand invasion and protospacer binding in V. cholerae. The published protospacer-adjacent motif (PAM), 5′-TT-3′, is shown in blue at the –1 and –2 positions. The 5′ spacer nucleotide is highlighted in red at the +1 position, where it complements the protospacer nucleotide (in green). (B) Effect of a transversion mutation at the +1 site. The *aad9* gene in pDL1301 was silently mutated at the +1 site from a purine to a pyrimidine, or vice versa, at targeting sites to create eight variations of donor plasmid (aad9*). The corresponding spacer in targeting strains was then changed to match each new donor plasmid so as to preserve base pairing at the +1 position, creating eight new targeting strains that individually pair to their donor strains (crRNA*). The conjugation efficiencies of these eight new pairs were obtained. Data for the original, unmodified conjugation (filled circles) are reproduced from Fig. 2 for the sake of comparison. The crRNA-PAM diagrams above the graph represent the modified condition. Data represent the mating efficiencies of three independently obtained V. cholerae exconjugates.

The results in Fig. 2 show that a pyrimidine in the +1 crRNA spacer position with a complementary purine in the protospacer is necessary for efficient protospacer targeting, which is shown schematically in Fig. 3A. However, several spacer attributes may influence targeting, including GC content, crRNA stability and processing, and other poorly understood parameters (26–28). To control for confounding intrinsic spacer attributes, the 5′ nucleotide in each pCRISPR targeting construct was mutated from a pyrimidine to a purine, or vice versa (spacer*). Then the target *aad9* gene in donor plasmid pDL1301 was individually and separately silently modified so that base pairing was restored at the +1 position with each new targeting spacer (aad9*). Thus, each *aad9* mutation additionally changes the putative targeting motif from 5′-RTT-3′ to 5′-YTT-3′, and vice versa. The new spacers, PAMs, and corresponding *aad9* mutations are listed in Table 2. The conjugation assay was then done on each matching pair of mutated pDL1301 and targeting strain, and the results were compared to data from Fig. 2 (Fig. 3B). Switching a 5′ pyrimidine spacer/5′ purine PAM pair to a purine/pyrimidine pair abolished targeting. In contrast, switching a 5′ purine spacer/5′ pyrimidine PAM pair to a pyrimidine/purine pair provided modest increases in interference for 5′-purine spacers 1 and 4, and substantial increases for spacers 2 and 3. In total, these results confirm the necessity of a 5′-pyrimidine in the +1 spacer position and/or an opposite complementary purine in the target for efficient CRISPR interference activity, independent of individual spacer characteristics.

**TABLE 2:** Modifications of targeting spacer and corresponding silent mutations in the *aad9* gene

Name	Targeting strand	aad9*	Modified PAM	Modified spacer sequence
Pyrimidine-1*	–1	A153T	5′-TTT-3′	5′-AGGTTCAGATACGACGACTAAAAAGTCAAGATC-3′
Pyrimidine-2*	1	C400A	5′-TTT-3′	5′-AGAAAAAATAAAAGAATATACGGAAATTATGAC-3′
Pyrimidine-3*	–1	A459T	5′-TTT-3′	5′-AGGAATATCAGGTAGTAATTCCTCTAAGTCATA-3′
Pyrimidine-4*	–1	A540T	5′-TTT-3′	5′-AATAGAGTTGGTTTCATCATCCTGATAATTATC-3′
Purine-1*	1	A412C	5′-GTT-3′	5′-CGAATATACGGAAATTATGACTTAGAGGAATTA-3′
Purine-2*	–1	T546A	5′-ATT-3′	5′-TGTTAATATAGAGTTGGTTTCATCATCCTGATA-3′
Purine-3*	–1	T561A	5′-ATT-3′	5′-TATCATACGGCATAAAGTTAATATAGAGTTGGT-3′
Purine-4*	–1	T651A	5′-ATT-3′	5′-TATTCTCTCCCTATGTTCTAATGGAGAAGATTC-3′

### Disruption of potential base pairing at the +1 position affects interference.

**FIG 4:**
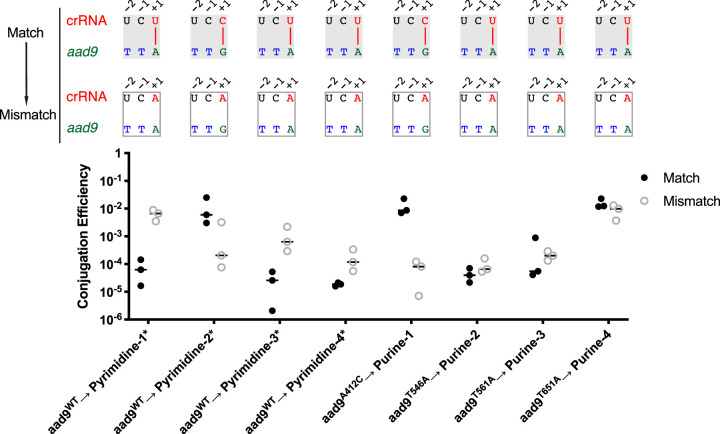
Effect of disallowing base pairing at the +1 PAM nucleotide position on interference activity. (Top) Mating modified 5′-pyrimidine* constructs with wild-type *aad9* (*aad9*^WT^) or mating PAM-modified pDL1301 into corresponding 5′-purine spacer constructs (aad9*) disrupts base pairing with PAM at the +1 position while preserving 5′-RTT-3′ PAM. The crRNA-PAM architecture of the original matched pairs preserving base pairing at the +1 position is shown above, with the architecture of the mismatched pairs below. Each match-mismatch pair corresponds to the conjugation efficiencies plotted at the bottom. (Bottom) Conjugation efficiencies of matings with mismatched pairs compared to those with matched pairs at the +1 position. Data for the conjugation efficiencies of *aad9*^WT^ into unmodified pyrimidine targeting strains (filled circles) are reproduced from Fig. 2 for the sake of comparison. Data for the matched conjugation pairs of mutated *aad9* with corresponding 5′-modified purine targeting strains (open circles) are reproduced from Fig. 3B for the sake of comparison. Data represent conjugation efficiencies from three independent experiments.

Thus far, our results have shown that, for effective interference, there must be a pyrimidine in the +1 spacer position complementary to a target purine. It is unclear which sequence requirement is dominant—that is, if the conserved spacer pyrimidine occurs as a consequence of a stringent purine requirement in the PAM, or vice versa. To independently examine the roles of the 5′ spacer pyrimidine and the 5′ PAM nucleotide in interference, unmodified pDL1301 was mated into 5′-pyrimidine mutated targeting constructs pyrimidine-1* through pyrimidine-4*, and the modified aad9* pDL1301 plasmids were mated into the respective unmodified targeting constructs purine-1 through purine-4. These combinations preserve a 5′-RTT-3′ PAM in the target plasmid in all cases but disrupt potential base pairing at the +1 PAM position, since the crRNA spacers contain 5′-purines (Fig. 4). The conjugation efficiencies of these matings were compared to the efficiencies obtained when mating combinations preserved potential base pairing (Fig. 4). We observed that of the eight constructs that target a 5′-RTT-3′ PAM, eight reduced conjugation efficiency by at least 10-fold, and five reduced conjugation efficiency by at least 1,000-fold (Fig. 3). Furthermore, targeting of two of the three 5′-RTT-3′ PAMs was increased from 10- to 1,000-fold when the 5′ spacer nucleotide was mutated from a cytidine to an adenosine (Fig. 4). In contrast, we observed that under the same conditions, none of the eight 5′-YTT-3′ PAMs were targeted, demonstrating that the effects of mutating the 5′ crRNA nucleotide are always less than the effects of mutating the PAM (Fig. 3B). Together, these results support the conclusion that in V. cholerae, the true PAM for interference is 5′-RTT-3′.

We found that in three of eight cases, a decrease in targeting efficiency was observed upon targeting of a 5′-RTT-3′ PAM with a 5′ spacer purine (Fig. 4). In contrast, two combinations, *aad9*^WT^ → pyrimidine-2* and *aad9*^T546A^ → purine-1*, showed the opposite effect: targeting worked approximately 100-fold better when the 5′ spacer nucleotide was mutated to a purine. In the latter cases, a 5′-GTT-3′ PAM was targeted, while in the former cases, the spacers targeted a 5′-ATT-3′ PAM. However, a 5′-GTT-3′ PAM was not functionally inferior to a 5′-ATT-3′ PAM; in the two cases with a 5′-GTT-3′ PAM, targeting was inefficient only when a cytidine was the 5′ spacer nucleotide, and upon its mutation to an adenosine, targeting was just as efficient as that observed with 5′-ATT-3′ PAMs (Fig. 4). Perhaps the 5′ spacer cytidine base pairs with the guanosine of the protospacer PAM, thereby interfering with its recognition. Alternatively, the 5′ spacer cytidine may interfere with a step prior to targeting, such as pre-crRNA folding or processing.

### A 5′ spacer cytidine interferes with targeting independently of its potential for base pairing with the PAM.

**FIG 5:**
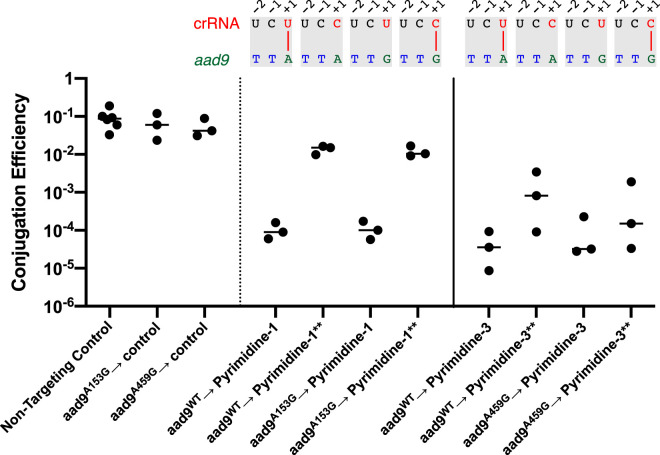
Effects of spacer nucleotide modifications targeting 5′-RTT-3′ PAMs. The +1 spacer nucleotide in targeting constructs pyrimidine-1 and pyrimidine-3 was modified from a T to a C (pyrimidine**), and the corresponding +1 PAM nucleotide in *aad9* was modified from an A to a G. All combinations of donor and recipient were then mated, and the conjugation efficiency was obtained. The corresponding crRNA-*aad9* architectures are shown above each result. Matings of each donor *aad9* plasmid to a nontargeting spacer were obtained as conjugation controls.

To further examine the targeting efficiency of 5′-GTT-3′ PAMs relative to 5′-ATT-3′ PAMs, as well as the importance of the 5′ spacer nucleotide in that process, the 5′ spacer nucleotides of each of the pCRISPR targeting constructs pyrimidine-1 and pyrimidine-3 were modified from a thymidine to a cytidine (pyrimidine-1**, pyrimidine-3**). The corresponding *aad9* protospacers for pyrimidine-1 and pyrimidine-3 were then silently modified to alter the PAM from 5′-ATT-3′ to 5′-GTT-3′, generating two new pDL1301 variants (*aad9*^A153G^, *aad9*^A456G^). The net result provides four possible donor-recipient pairings for each spacer context in which the 5′ crRNA uridine or cytidine each targets either a 5′-ATT-3′ or a 5′-GTT-3′ PAM (Fig. 5). The conjugation efficiencies were then obtained for all potential pairings as described above.

In the case of the first protospacer, targeting was highly efficient regardless of whether the PAM was 5′-ATT-3′ or 5′-GTT-3, so long as the targeting spacer contained a 5′ uridine. However, for both PAMs, when the 5′ spacer nucleotide was mutated to a cytidine, the targeting efficiency dropped by more than 2 orders of magnitude. With the third protospacer, although the magnitude of the effects was less dramatic, the same trends were observed. We note that for both the first and third protospacers, the inhibitory effect of a 5′ spacer cytidine on targeting could be overcome upon overexpression of both crRNA and *cas* proteins by IPTG (Fig. S1). A spacer with a cytidine at its 5′ end is still impaired for targeting but lacks the potential for base pairing with a 5′-ATT-3′ PAM. Therefore, we can rule out any model that invokes the importance of base pairing with the PAM in that process. Thus, in the V. cholerae type I-E CRISPR/Cas system, it is likely that a 5′ spacer cytidine inhibits targeting at a step prior to protospacer interaction, such as impairing the folding and/or processing of the precursor crRNA.

10.1128/mSphere.00813-20.1 FIG S1 Effects of spacer nucleotide modifications targeting 5′-RTT-3′ PAMs in the presence of 100 μM IPTG. The +1 spacer nucleotide in targeting constructs pyrimidine-1 and pyrimidine-3 was modified from a T to a C (pyrimidine**), and the corresponding +1 PAM nucleotide in *aad9* was modified from an A to a G. All combinations of donor and recipient were then mated, and the conjugation efficiency was obtained. The corresponding crRNA-*aad9* architectures are shown above each result. Matings of each donor *aad9* plasmid to a nontargeting spacer were obtained as conjugation controls. Download FIG S1, PDF file, 0.2 MB. Copyright © 2020 Bourgeois et al. 2020 Bourgeois et al. This content is distributed under the terms of the Creative Commons Attribution 4.0 International license.

## DISCUSSION

The molecular history recorded in the CRISPR/Cas regions of bacterial genomes is a valuable resource for researchers studying molecular epidemiology and encounters with foreign DNA. Numerous databases and Web servers have been developed to detect CRISPR arrays and Cas gene clusters (29, 30). We opted for a straightforward approach to obtain spacer content from arrays across multiple isolates of V. cholerae. Our script allows us to automate spacer extraction, protospacer/PAM detection, and extraction of annotation information from the target regions to assist in analysis. The running parameters are easily modifiable to permit mining of the spacer, protospacer, and PAM-associated information of any bacterial species with any known repeat sequence.

Horizontal transfer of mobile genetic elements has played a key role in the success of V. cholerae as a human pathogen. For example, genes encoding cholera toxin are carried by the lysogenic bacteriophage CTXΦ (31), and the integrative conjugatable element SXT provides resistance to multiple antibiotics (32). Many isolates with detected CRISPR repeats do not possess these and other key virulence factors necessary for human infection. One hypothesis is that CRISPR/Cas provides a barrier to the acquisition of advantageous traits that aid infection. CRISPR/Cas is negatively associated with virulence in E. coli and negatively impacts natural transformation in Streptococcus pneumoniae (33–35). In agreement with this, we discovered spacers against several plasmids and filamentous CTXΦ-like prophages. We also found spacers targeting prophage genes homologous to *rstA2* and *rstB2* of RS2-CTXΦ, genes that are necessary for the replication and integration of CTXΦ (36). Increased exposure in V. cholerae of the El Tor biotype to incoming genetic elements that provide selective advantages may outweigh any disadvantages that occur from the loss of adaptive immunity directed against predatory phages (37, 38). Indeed, some V. cholerae strains have acquired specific genomic islands, termed PLEs (phage-inducible chromosomal island-like elements), that inhibit the replication of the ICP1 vibriophage and thereby obviate CRISPR/Cas in that setting (39).

The CRISPR/Cas system in V. cholerae was thought to be limited to strains of the classical biotype. Since this biotype is thought to be extinct (12, 40, 41), it was assumed that its CRISPR/Cas system was also extinct. Here, we found direct evidence to the contrary, as we identified multiple environmental strains isolated in the past 3 years from locations as diverse as Bangladesh, Russia, and Australia, all containing the CRISPR/Cas system. Hence, the system continues to impact the evolution of V. cholerae.

The type I-E CRISPR/Cas system in E. coli is most commonly described as having 32-bp spacer sequences flanked by 29-bp repeats in its CRISPR array, targeting a 3-bp 5′-CWT-3′ PAM (22, 42). In the crRNA of this system, the terminal guanosine that could potentially base pair with the cytidine of the 5′-CWT-3′ PAM was initially assumed to be part of the repeat and to have been created by repeat duplication during acquisition (43). However, it has been shown that this guanosine is instead captured from incoming prespacers of foreign DNA (9, 22, 44). In V. cholerae, a pyrimidine in the CRISPR array occupies the position analogous to that of the guanosine in the E. coli system. In nearly all mined protospacers, when the array pyrimidine was a cytidine, a guanosine was observed in the protospacer PAM, while a thymidine in the array was almost always associated with an adenosine. Furthermore, within individual CRISPR arrays, 5′-thymidine and 5′-cytidine spacers are randomly distributed. Together, these results indicate that the pyrimidine does not result from repeat duplication during spacer integration and is instead protospacer derived. Our observations in V. cholerae support findings in the analogous E. coli system, namely, that in the E. coli system, the guanosine is similarly protospacer derived and should be considered the first position of the spacer. By that definition, as is the case for V. cholerae, the system has 33-bp spacer sequences flanked by 28-bp repeats.

Protospacer-adjacent motifs (PAMs) provide at least three functions: they are required for capture and adaptation, they are required for interference, and they are required for self-discrimination (10). Here, we propose that the PAM for the V. cholerae system is 5′-RTT-3′. The bioinformatic data presented support the importance of the 5′ purine in acquisition, while experimental data support its importance in interference. However, this nucleotide is not adjacent to the protospacer and is instead captured as the prespacer terminal nucleotide. In the majority of type I and type II CRISPR/Cas systems, this corresponding +1 PAM position is not retained in the captured spacer following target cleavage (8, 45–47). We propose that in the V. cholerae system, cleavage occurs within the PAM during capture, thereby incorporating a single complementary nucleotide into its spacer. In this context, the PAM might better be referred to as a protospacer-associated motif to reflect its origin and function (10).

Base pairing between spacer and protospacer sequences is crucial for interference (48). Since the +1 PAM nucleotide is derived from the protospacer, and the complementary pyrimidine is captured in the CRISPR array, the potential for base pairing between the PAM and the 5′ nucleotide of the crRNA spacer appears inevitable. However, in E. coli, any potential for base pairing between the 5′ crRNA nucleotide and cognate PAM nucleotide in the phage M13 g8 spacer-protospacer pairing is dispensable for interference (49). More recently, a crystal structure of E. coli Cascade bound to target double-stranded DNA (dsDNA) revealed that the 5′ crRNA guanosine and the +1 PAM cytidine are not associated; instead, the target PAM DNA remains paired to the nontarget strand (50). In V. cholerae, we found that both 5′-GTT-3′ and 5′-ATT-3′ PAMs could be targeted with similar efficiency. Unexpectedly, we also demonstrate large changes in interference when the 5′ crRNA nucleotide is mutated. In particular, we observed that a 5′-spacer cytidine prevented efficient interference irrespective of whether that cytidine had the potential to pair with the cognate position in the PAM. Such a result is consistent with the lack of pairing observed between these nucleotides in the E. coli system. Therefore, in V. cholerae, the 5′-spacer cytidine probably interferes with a process prior to interaction with the protospacer, such as precursor crRNA folding and/or processing. Based on our current results, for ideal spacer design, one should search for a 5′-AAYN_32_-3′ sequence motif in target DNA and incorporate 5′-TN_32_-3′ into the pCRISPR spacer-expressing plasmid. TN_32_ sequences with the potential to form a stable RNA structure should be avoided, since such structures could impair processing and/or protospacer annealing (26–28).

The finding that spacers with a 5′ cytidine perform worse than those with 5′ thymidine is surprising, given that according to the bioinformatic data, the two are captured at approximately the same frequency (Fig. 1D). It is possible that, analogously to what we observed upon induction of the experimental system (Fig. S1), the level of expression of the natural CRISPR machinery exceeds that of the uninduced experimental system and thereby rescues the effects of the 5′ cytidine phenotype. One argument against this is that 5′-YTT-3′ PAMs are almost never observed in nature (Fig. 1D), yet in the experimental system, induction fully rescued targeting of two of the four 5′-YTT-3′ PAMs tested (Fig. 2). Hence, the level of expression of the induced experimental system likely exceeds that of the natural system, and it remains possible that in natural systems, 5′ thymidine spacers outperform those with 5′ cytidine. A direct experimental comparison of each spacer type in a natural system will be needed to resolve this issue.

In conclusion, we found evidence that the CRISPR/Cas system originally observed in an extinct strain of V. cholerae continues to be present in other strains today. By mining deposited sequences, we identified a conserved 5′-pyrimidine in naturally occurring spacers and found that their cognate protospacers contain a complementary purine. This purine was found to be essential for interference when crRNA and Cas proteins are not overexpressed; thus, we have redefined the PAM of the system as 5′-RTT-3′. Finally, we demonstrated that interference is affected by the identity of the 5′ pyrimidine, where cytidines hamper efficient targeting. Our findings further the understanding of the molecular mechanisms of type I-E CRISPR/Cas systems. The resulting enhancement in our knowledge of the requirements for interference should allow for more-intelligent design of targeting spacers and permit efficient utilization of CRISPR in genomic manipulation of V. cholerae and its phages.

## MATERIALS AND METHODS

### Bacterial strains and culture conditions.

The bacterial strains and plasmids used in this study are listed in Table 3. Bacteria were cultivated at 37°C in Luria Broth (LB) agar or in LB. The medium was supplemented with ampicillin (Amp; 50 μg/ml), kanamycin (Kan; 50 μg/ml), spectinomycin (Spec; 100 μg/ml), and/or streptomycin (Sm; 100 μg/ml) when appropriate. For induction, LB agar plates were supplemented with 100 μM isopropyl-β-d-thiogalactopyranoside (IPTG).

**TABLE 3:** Bacterial strains and plasmids used in this study

Strain or plasmid	Description	Source or reference
V. cholerae strains		
E7946	El Tor biotype, serogroup O1; Sm^r^	52
KS916	E7946 with CRISPR/Cas array from O395 under the control of the *tac* promoter; Kan^r^ Δ*lacZ*::Spec	15
AC6625	KS916, wild-type *lacZ* by natural transformation of *lacZ* locus from E7946; Kan^r^	This study
Plasmids		
pCRISPR	Expression plasmid containing the V. cholerae CRISPR spacer array under the control of the *tac* promoter in the pMMB67EH background; Amp^r^	15
pDL1301	Conjugation plasmid with p15a minimal origin, RP4 *oriT*, and spectinomycin adenyltransferase gene *aad9*; Spec^r^	This study

### Bioinformatic analysis of CRISPR repeats in deposited sequences of V. cholerae.

Additional strains containing the 28-bp V. cholerae O395 CRISPR repeat (5′-GTCTTCCCCACGCAGGTGGGGGTGTTTC-3′) were identified using BLAST to search the NCBI whole-genome shotgun contigs database, restricting results to the family *Vibrionaceae*. This method would identify O395 CRISPR repeats when present in other *Vibrio* species; however, we found that only V. cholerae strains contained the perfect repeat. Extraction of spacer content and protospacer mining were automated using a custom Python script (https://github.com/camillilab/spacer_miner). Briefly, identified contigs containing repeat sequences were retrieved from NCBI. Spacers were extracted if they had sequences no longer than 50 nucleotides, were flanked by perfect repeat sequences, and did not contain ambiguous nucleotides. Each unique spacer was then compared with BLAST to the NCBI nonredundant nucleotide database in order to identify putative protospacers. Putative protospacers were considered if there was at least 93% identity to the corresponding spacer over 96% of the spacer sequence, which permits up to two mismatches and one missing or additional base in the protospacer hit. Sequence logos were generated using unique spacers and nucleotides 3′ of identified protospacers using WebLogo (51).

### Conjugation assays.

The donor plasmid pDL1301, an RP4-conjugatable plasmid that confers resistance to spectinomycin, and its variants were transferred using E. coli SM10λpir. Donor and recipient AC6625 strains were grown to an optical density at 600 nm ( OD_600_) of 1.0. Then 500 μl of the donor and 500 μl of the recipient were pelleted, washed once in phosphate-buffered saline (PBS), and resuspended in 50 μl PBS. A 1:1 mixture was applied to a sterile filter (pore size, 0.22 μm; Millipore) on an LB plate and was incubated at 37°C for 2 h. Bacteria were recovered from the filter by vortexing in 500 μl PBS. Serial dilutions were plated onto a medium selective for recipient V. cholerae and exconjugates and were plated separately in the presence of IPTG. The conjugation efficiency was calculated as the number of exconjugates divided by the total number of viable V. cholerae recipients.

### Construction of targeting plasmids.

CRISPR targeting plasmids were generated using the previously constructed pCRISPR backbone (15). New spacers were constructed using annealed and phosphorylated oligonucleotides that included the targeting region flanked by appropriate 5′ and 3′ overhangs to facilitate ligation into pCRISPR. The resulting double-stranded DNAs were cloned into pCRISPR by Golden Gate cloning using BsaI-HF (New England Biolabs). Ligation products were purified and used to transform electrocompetent E. coli SM10λpir. DNA from individual clones was isolated, and the targeting sequence was verified by Sanger sequencing. These plasmids were then mated into the V. cholerae targeting strain AC6625.

### Generation of mutant donor and targeting plasmids.

The target *aad9* gene in donor plasmid pDL1301 was modified in eight independent sites by site-directed PCR mutagenesis to yield eight new donor plasmids possessing one silent mutation each (aad9*). The location of each of these mutations is listed in Table 2. Eight new spacers that target these mutated sites were introduced into targeting plasmid pCRISPR as previously described to yield eight new targeting plasmids (crRNA*). pCRISPR constructs pyrimidine-1** and pyrimidine-3** and pDL1301 constructs *aad9*^A153G^ and *aad9*^A456G^ were constructed in the same manner.

## References

[1] Barrangou R, Fremaux C, Deveau H, Richards M, Boyaval P, Moineau S, Romero DA, Horvath P 2007 CRISPR provides acquired resistance against viruses in prokaryotes. Science 315:1709–1712. doi:10.1126/science.1138140. 17379808

[2] Samai P, Pyenson N, Jiang W, Goldberg GW, Hatoum-Aslan A, Marraffini LA 2015 Co-transcriptional DNA and RNA cleavage during type III CRISPR-Cas immunity. Cell 161:1164–1174. doi:10.1016/j.cell.2015.04.027. 25959775 PMC4594840

[3] Koonin EV, Makarova KS, Zhang F 2017 Diversity, classification and evolution of CRISPR-Cas systems. Curr Opin Microbiol 37:67–78. doi:10.1016/j.mib.2017.05.008. 28605718 PMC5776717

[4] Jackson SA, McKenzie RE, Fagerlund RD, Kieper SN, Fineran PC, Brouns SJ 2017 CRISPR-Cas: adapting to change. Science 356:eaal5056. doi:10.1126/science.aal5056. 28385959

[5] Makarova KS, Haft DH, Barrangou R, Brouns SJ, Charpentier E, Horvath P, Moineau S, Mojica FJ, Wolf YI, Yakunin AF, van der Oost J, Koonin EV 2011 Evolution and classification of the CRISPR-Cas systems. Nat Rev Microbiol 9:467–477. doi:10.1038/nrmicro2577. 21552286 PMC3380444

[6] van der Oost J, Westra ER, Jackson RN, Wiedenheft B 2014 Unravelling the structural and mechanistic basis of CRISPR-Cas systems. Nat Rev Microbiol 12:479–492. doi:10.1038/nrmicro3279. 24909109 PMC4225775

[7] Leenay RT, Maksimchuk KR, Slotkowski RA, Agrawal RN, Gomaa AA, Briner AE, Barrangou R, Beisel CL 2016 Identifying and visualizing functional PAM diversity across CRISPR-Cas systems. Mol Cell 62:137–147. doi:10.1016/j.molcel.2016.02.031. 27041224 PMC4826307

[8] Mojica FJM, Diez-Villasenor C, Garcia-Martinez J, Almendros C 2009 Short motif sequences determine the targets of the prokaryotic CRISPR defence system. Microbiology (Reading) 155:733–740. doi:10.1099/mic.0.023960-0. 19246744

[9] Yosef I, Goren MG, Qimron U 2012 Proteins and DNA elements essential for the CRISPR adaptation process in *Escherichia coli*. Nucleic Acids Res 40:5569–5576. doi:10.1093/nar/gks216. 22402487 PMC3384332

[10] Shah SA, Erdmann S, Mojica FJ, Garrett RA 2013 Protospacer recognition motifs: mixed identities and functional diversity. RNA Biol 10:891–899. doi:10.4161/rna.23764. 23403393 PMC3737346

[11] Deveau H, Barrangou R, Garneau JE, Labonte J, Fremaux C, Boyaval P, Romero DA, Horvath P, Moineau S 2008 Phage response to CRISPR-encoded resistance in *Streptococcus thermophilus*. J Bacteriol 190:1390–1400. doi:10.1128/JB.01412-07. 18065545 PMC2238228

[12] Harris JB, LaRocque RC, Qadri F, Ryan ET, Calderwood SB 2012 Cholera. Lancet 379:2466–2476. doi:10.1016/S0140-6736(12)60436-X. 22748592 PMC3761070

[13] Sack DA, Sack RB, Nair GB, Siddique AK 2004 Cholera. Lancet 363:223–233. doi:10.1016/s0140-6736(03)15328-7. 14738797

[14] Chun J, Grim CJ, Hasan NA, Lee JH, Choi SY, Haley BJ, Taviani E, Jeon YS, Kim DW, Lee JH, Brettin TS, Bruce DC, Challacombe JF, Detter JC, Han CS, Munk AC, Chertkov O, Meincke L, Saunders E, Walters RA, Huq A, Nair GB, Colwell RR 2009 Comparative genomics reveals mechanism for short-term and long-term clonal transitions in pandemic *Vibrio cholerae*. Proc Natl Acad Sci U S A 106:15442–15447. doi:10.1073/pnas.0907787106. 19720995 PMC2741270

[15] Box AM, McGuffie MJ, O'Hara BJ, Seed KD 2016 Functional analysis of bacteriophage immunity through a type I-E CRISPR-Cas system in *Vibrio cholerae* and its application in bacteriophage genome engineering. J Bacteriol 198:578–590. doi:10.1128/JB.00747-15. 26598368 PMC4719448

[16] Hatoum-Aslan A 2018 Phage genetic engineering using CRISPR^–^Cas systems. Viruses 10:335. doi:10.3390/v10060335. PMC6024849 29921752

[17] Chen Y, Batra H, Dong J, Chen C, Rao VB, Tao P 2019 Genetic engineering of bacteriophages against infectious diseases. Front Microbiol 10:954. doi:10.3389/fmicb.2019.00954. 31130936 PMC6509161

[18] Kiro R, Shitrit D, Qimron U 2014 Efficient engineering of a bacteriophage genome using the type I-E CRISPR-Cas system. RNA Biol 11:42–44. doi:10.4161/rna.27766. 24457913 PMC3929423

[19] Martel B, Moineau S 2014 CRISPR-Cas: an efficient tool for genome engineering of virulent bacteriophages. Nucleic Acids Res 42:9504–9513. doi:10.1093/nar/gku628. 25063295 PMC4132740

[20] Bourgeois J, Lazinski DW, Camilli A 2020 Identification of spacer and protospacer sequence requirements in the *Vibrio cholerae* type I-E CRISPR/Cas system. bioRxiv doi:10.1101/2020.08.09.243105. PMC7677007 33208517

[21] Sobecky PA, Mincer TJ, Chang MC, Toukdarian A, Helinski DR 1998 Isolation of broad-host-range replicons from marine sediment bacteria. Appl Environ Microbiol 64:2822–2830. doi:10.1128/AEM.64.8.2822-2830.1998. 9687436 PMC106778

[22] Wang J, Li J, Zhao H, Sheng G, Wang M, Yin M, Wang Y 2015 Structural and mechanistic basis of PAM-dependent spacer acquisition in CRISPR-Cas systems. Cell 163:840–853. doi:10.1016/j.cell.2015.10.008. 26478180

[23] de Boer HA, Comstock LJ, Vasser M 1983 The *tac* promoter: a functional hybrid derived from the *trp* and *lac* promoters. Proc Natl Acad Sci U S A 80:21–25. doi:10.1073/pnas.80.1.21. 6337371 PMC393301

[24] Xue C, Seetharam AS, Musharova O, Severinov K, Brouns SJ, Severin AJ, Sashital DG 2015 CRISPR interference and priming varies with individual spacer sequences. Nucleic Acids Res 43:10831–10847. doi:10.1093/nar/gkv1259. 26586800 PMC4678831

[25] Karvelis T, Gasiunas G, Young J, Bigelyte G, Silanskas A, Cigan M, Siksnys V 2015 Rapid characterization of CRISPR-Cas9 protospacer adjacent motif sequence elements. Genome Biol 16:253. doi:10.1186/s13059-015-0818-7. 26585795 PMC4653880

[26] Doench JG, Hartenian E, Graham DB, Tothova Z, Hegde M, Smith I, Sullender M, Ebert BL, Xavier RJ, Root DE 2014 Rational design of highly active sgRNAs for CRISPR-Cas9-mediated gene inactivation. Nat Biotechnol 32:1262–1267. doi:10.1038/nbt.3026. 25184501 PMC4262738

[27] Chari R, Mali P, Moosburner M, Church GM 2015 Unraveling CRISPR-Cas9 genome engineering parameters via a library-on-library approach. Nat Methods 12:823–826. doi:10.1038/nmeth.3473. 26167643 PMC5292764

[28] Chari R, Yeo NC, Chavez A, Church GM 2017 sgRNA Scorer 2.0: a species-independent model to predict CRISPR/Cas9 activity. ACS Synth Biol 6:902–904. doi:10.1021/acssynbio.6b00343. 28146356 PMC5793212

[29] Grissa I, Vergnaud G, Pourcel C 2007 The CRISPRdb database and tools to display CRISPRs and to generate dictionaries of spacers and repeats. BMC Bioinformatics 8:172. doi:10.1186/1471-2105-8-172. 17521438 PMC1892036

[30] Couvin D, Bernheim A, Toffano-Nioche C, Touchon M, Michalik J, Neron B, Rocha EPC, Vergnaud G, Gautheret D, Pourcel C 2018 CRISPRCasFinder, an update of CRISRFinder, includes a portable version, enhanced performance and integrates search for Cas proteins. Nucleic Acids Res 46:W246–W251. doi:10.1093/nar/gky425. 29790974 PMC6030898

[31] Waldor MK, Mekalanos JJ 1996 Lysogenic conversion by a filamentous phage encoding cholera toxin. Science 272:1910–1914. doi:10.1126/science.272.5270.1910. 8658163

[32] Waldor MK, Tschape H, Mekalanos JJ 1996 A new type of conjugative transposon encodes resistance to sulfamethoxazole, trimethoprim, and streptomycin in *Vibrio cholerae* O139. J Bacteriol 178:4157–4165. doi:10.1128/jb.178.14.4157-4165.1996. 8763944 PMC178173

[33] Bikard D, Hatoum-Aslan A, Mucida D, Marraffini LA 2012 CRISPR interference can prevent natural transformation and virulence acquisition during *in vivo* bacterial infection. Cell Host Microbe 12:177–186. doi:10.1016/j.chom.2012.06.003. 22901538

[34] Garcia-Gutierrez E, Almendros C, Mojica FJ, Guzman NM, Garcia-Martinez J 2015 CRISPR content correlates with the pathogenic potential of *Escherichia coli*. PLoS One 10:e0131935. doi:10.1371/journal.pone.0131935. 26136211 PMC4489801

[35] Toro M, Cao G, Ju W, Allard M, Barrangou R, Zhao S, Brown E, Meng J 2014 Association of clustered regularly interspaced short palindromic repeat (CRISPR) elements with specific serotypes and virulence potential of Shiga toxin-producing *Escherichia coli*. Appl Environ Microbiol 80:1411–1420. doi:10.1128/AEM.03018-13. 24334663 PMC3911044

[36] Waldor MK, Rubin EJ, Pearson GD, Kimsey H, Mekalanos JJ 1997 Regulation, replication, and integration functions of the *Vibrio cholerae* CTXΦ are encoded by region RS2. Mol Microbiol 24:917–926. doi:10.1046/j.1365-2958.1997.3911758.x. 9220000

[37] Nelson EJ, Harris JB, Morris JG Jr, Calderwood SB, Camilli A 2009 Cholera transmission: the host, pathogen and bacteriophage dynamic. Nat Rev Microbiol 7:693–702. doi:10.1038/nrmicro2204. 19756008 PMC3842031

[38] Faruque SM, Mekalanos JJ 2012 Phage-bacterial interactions in the evolution of toxigenic *Vibrio cholerae*. Virulence 3:556–565. doi:10.4161/viru.22351. 23076327 PMC3545932

[39] O'Hara BJ, Barth ZK, McKitterick AC, Seed KD 2017 A highly specific phage defense system is a conserved feature of the *Vibrio cholerae* mobilome. PLoS Genet 13:e1006838. doi:10.1371/journal.pgen.1006838. 28594826 PMC5481146

[40] Faruque SM, Abdul Alim AR, Rahman MM, Siddique AK, Sack RB, Albert MJ 1993 Clonal relationships among classical *Vibrio cholerae* O1 strains isolated between 1961 and 1992 in Bangladesh. J Clin Microbiol 31:2513–2516. doi:10.1128/JCM.31.9.2513-2516.1993. 7691878 PMC265789

[41] Alam M, Islam MT, Rashed SM, Johura FT, Bhuiyan NA, Delgado G, Morales R, Mendez JL, Navarro A, Watanabe H, Hasan NA, Colwell RR, Cravioto A 2012 *Vibrio cholerae* classical biotype strains reveal distinct signatures in Mexico. J Clin Microbiol 50:2212–2216. doi:10.1128/JCM.00189-12. 22518867 PMC3405568

[42] Musharova O, Sitnik V, Vlot M, Savitskaya E, Datsenko KA, Krivoy A, Fedorov I, Semenova E, Brouns SJJ, Severinov K 2019 Systematic analysis of type I-E *Escherichia coli* CRISPR-Cas PAM sequences ability to promote interference and primed adaptation. Mol Microbiol 111:1558–1570. doi:10.1111/mmi.14237. 30875129 PMC6568314

[43] Nunez JK, Lee AS, Engelman A, Doudna JA 2015 Integrase-mediated spacer acquisition during CRISPR-Cas adaptive immunity. Nature 519:193–198. doi:10.1038/nature14237. 25707795 PMC4359072

[44] Goren MG, Yosef I, Auster O, Qimron U 2012 Experimental definition of a clustered regularly interspaced short palindromic duplicon in *Escherichia coli*. J Mol Biol 423:14–16. doi:10.1016/j.jmb.2012.06.037. 22771574

[45] Erdmann S, Garrett RA 2012 Selective and hyperactive uptake of foreign DNA by adaptive immune systems of an archaeon via two distinct mechanisms. Mol Microbiol 85:1044–1056. doi:10.1111/j.1365-2958.2012.08171.x. 22834906 PMC3468723

[46] Nunez JK, Harrington LB, Kranzusch PJ, Engelman AN, Doudna JA 2015 Foreign DNA capture during CRISPR-Cas adaptive immunity. Nature 527:535–538. doi:10.1038/nature15760. 26503043 PMC4662619

[47] Wilkinson M, Drabavicius G, Silanskas A, Gasiunas G, Siksnys V, Wigley DB 2019 Structure of the DNA-bound spacer capture complex of a type II CRISPR-Cas system. Mol Cell 75:90–101.e5. doi:10.1016/j.molcel.2019.04.020. 31080012 PMC6620040

[48] Semenova E, Jore MM, Datsenko KA, Semenova A, Westra ER, Wanner B, van der Oost J, Brouns SJ, Severinov K 2011 Interference by clustered regularly interspaced short palindromic repeat (CRISPR) RNA is governed by a seed sequence. Proc Natl Acad Sci U S A 108:10098–10103. doi:10.1073/pnas.1104144108. 21646539 PMC3121866

[49] Westra ER, Semenova E, Datsenko KA, Jackson RN, Wiedenheft B, Severinov K, Brouns SJ 2013 Type I-E CRISPR-Cas systems discriminate target from non-target DNA through base pairing-independent PAM recognition. PLoS Genet 9:e1003742. doi:10.1371/journal.pgen.1003742. 24039596 PMC3764190

[50] Hayes RP, Xiao Y, Ding F, van Erp PB, Rajashankar K, Bailey S, Wiedenheft B, Ke A 2016 Structural basis for promiscuous PAM recognition in type I-E Cascade from *E. coli*. Nature 530:499–503. doi:10.1038/nature16995. 26863189 PMC5134256

[51] Crooks GE, Hon G, Chandonia JM, Brenner SE 2004 WebLogo: a sequence logo generator. Genome Res 14:1188–1190. doi:10.1101/gr.849004. 15173120 PMC419797

[52] Levine MM, Black RE, Clements ML, Cisneros L, Saah A, Nalin DR, Gill DM, Craig JP, Young CR, Ristaino P 1982 The pathogenicity of nonenterotoxigenic *Vibrio cholerae* serogroup O1 biotype El Tor isolated from sewage water in Brazil. J Infect Dis 145:296–299. doi:10.1093/infdis/145.3.296. 7061878

