# Supplementary figures and images for: Identification of Spacer and Protospacer Sequence Requirements in the Vibrio cholerae Type I-E CRISPR/Cas System
Source: mSphere. 2020 Nov 18;5(6):e00813-20. doi: 10.1128/mSphere.00813-20 (PMC7677007; doi:10.1128/mSphere.00813-20)

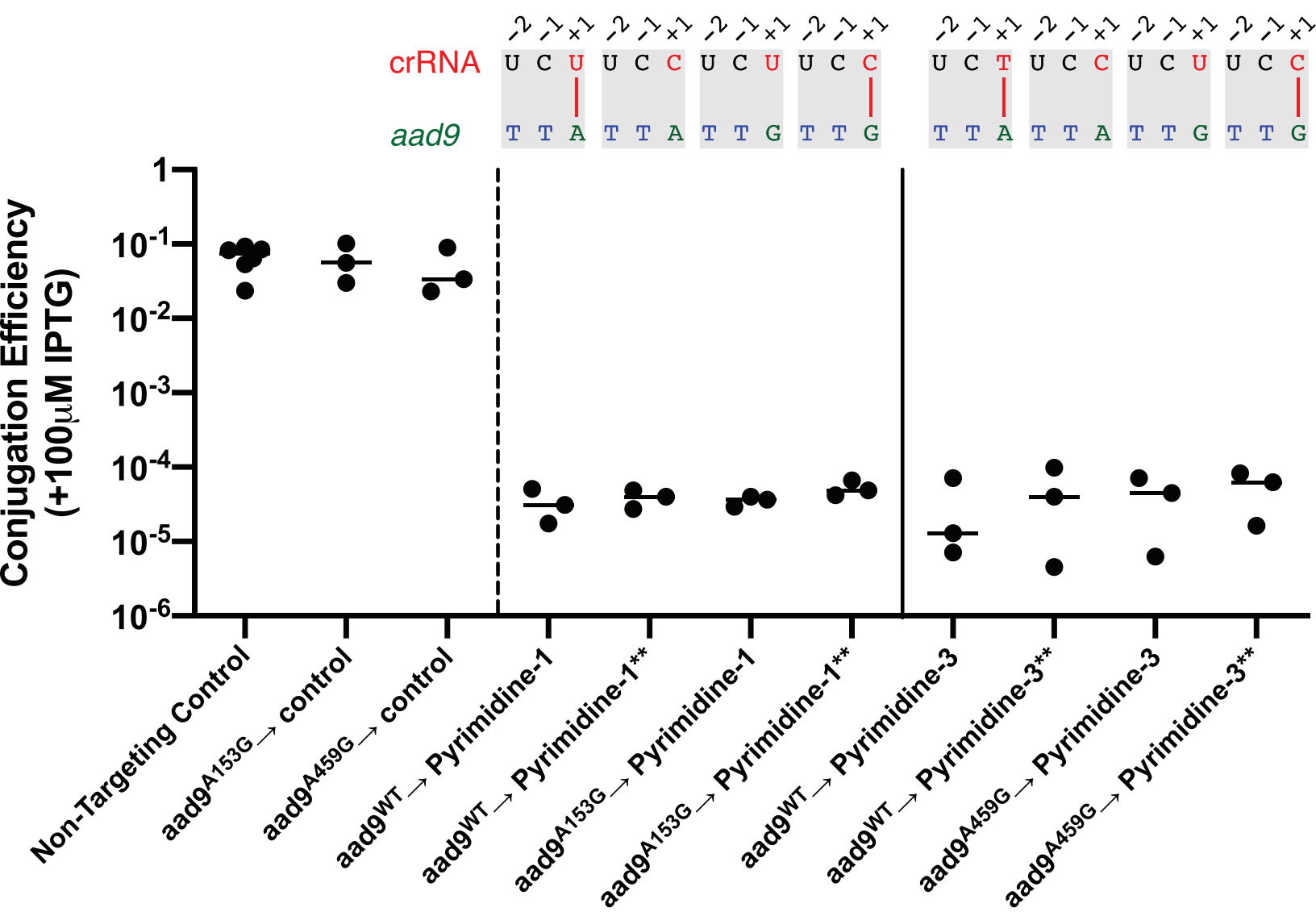

Supplement: FIG S1 [file mSphere.00813-20-sf001.pdf]
